# Fear of disease progression and relapse in multiple sclerosis: a systematic scoping review

**DOI:** 10.3389/fpsyt.2025.1680781

**Published:** 2025-11-12

**Authors:** Arashk Mallahzadeh, Tahereh Fereydoonnezhad, Lynne V. Gauthier, Mohsen Farjoud Kouhanjani, Shima Hosseinizadeh, Ali Khatibi

**Affiliations:** 1 School of Medicine, Shiraz University of Medical Sciences, Shiraz, Iran; 2 Clinical Neurology Research Center, Shiraz University of Medical Sciences, Shiraz, Iran; 3 Student Research Committee, Bushehr University of Medical Sciences, Bushehr, Iran; 4 Department of Physical Therapy and Kinesiology, University of Massachusetts Lowell, Lowell, MA, United States; 5 Department of Neurology, Epilepsy Center Frankfurt Rhein-Main, University Medicine Frankfurt, Goethe University Frankfurt, Frankfurt, Germany; 6 Department of Psychology, University of Bath, Bath, United Kingdom

**Keywords:** multiple sclerosis, fear of relapse, fear of progression, fear of disability, scoping review

## Abstract

**Background:**

Multiple sclerosis (MS) is a chronic disease of the central nervous system, most commonly affecting individuals in early adulthood. Uncertainty about future relapses and accumulating disability may lead to significant psychological distress, contributing to reduced quality of life.

**Objective:**

This systematic scoping review aimed to synthesize current evidence on the prevalence, determinants, and consequences of fear of relapse and disease progression in people living with MS.

**Method:**

The current systematic scoping review followed PRISMA-ScR guidelines. PubMed, Scopus, and Web of Science were searched from inception until October 6^th^. Original, English-language studies investigating fear of disease progression or relapse among people with multiple sclerosis were included, without restrictions on age, gender, disease stage, or study design. Data items were collected, and a quality assessment was conducted.

**Results:**

Our search yielded 43 studies, of which 13 met the eligibility criteria. Nine studies utilized the fear of relapse scale and the remaining four used the fear of progression questionnaire. Fear of progression among MS patients ranged from minimal to severe, with concerns centered on losing independence, relying on external help, and potential medication side effects. In relapsing-remitting MS, fear was particularly associated with uncertainty about disease course, potential disability, and worsening fatigue. Increased fear of relapse and progression was strongly associated with higher levels of fatigue, depression, stress, health anxiety, and lower quality of life.

**Conclusion:**

Fear of relapse and progression is common and significantly impacts the lives of people with MS. Future research should focus on evaluating and implementing tailored interventions — including psychological support, educational initiatives, and coping-based strategies — to help patients manage these fears.

## 1 Introduction

Multiple sclerosis (MS) is a chronic autoimmune disease of the central nervous system where immune cells attack the myelin sheath covering neurons (1). Globally, it is estimated that about 1.89 million people are living with MS, with more than 62,000 new cases diagnosed in 2021. The global prevalence is approximately 23.9 per 100,000 population, and this figure has shown a steady increase over the past three decades, reflecting both improved detection and a rising disease burden (2). MS typical onset is between 20–30 years of age and MS predominantly affects females compared to males [3:1 ratio] (3). Common symptoms include fatigue, visual disturbances, pain, and sensorimotor deficits (4). However, the disease course is highly variable. Some individuals experience long periods of remission with minimal symptoms, while others develop severe and progressive disability (5). The phenotype of the disease also varies between patients with the majority (85%) having the relapse remitting form (RRMS) followed by the primary progressive (PPMS) and the secondary progressive (SPMS) form (6). A relapse involves a flare-up of existing symptoms or the development of new ones during a phase of acute disease activity that lasts for over 24 hours that significantly alters a patients physical and/or mental health (7–9). Beyond its physical manifestations, MS has a profound psychological impact. Uncertainty about disease prognosis and the severity of future relapses often leads to significant fear of progression (10). This fear of progression (11) is related to distress, depression, generalized anxiety disorder, and reduced cognitive functioning (12) and adversely impacts quality of life (13, 14). Anxiety is common in people with MS, and fear is a central component of the anxious thoughts experienced by patients with chronic illnesses (15, 16). Due to the substantial contribution of fear of relapse and disease progression to the quality of life of people with MS, a systematical investigation of existing evidence offers critical insights into the prevalence, associated factors, and impacts of this fear in individuals with MS. However, despite growing recognition of its importance, recent studies remain fragmented, often focusing on anxiety or quality of life more broadly rather than specifically addressing fear of relapse and progression as a distinct construct. Moreover, there is no comprehensive synthesis of the literature that examines its prevalence, determinants, and consequences in one place.

While fear of disease progression has been explored extensively in other chronic illnesses such as cancer (17), it remains relatively underexamined in the context of MS.

To our knowledge, this is the first systematic review to address the burden of fear of relapse and disease progression in MS. Understanding how fear of disease relapse and progression affects quality of life can inform future tailored interventions for over 2.8 million people living with MS worldwide (18). This systematic scoping review summarizes the current literature on the prevalence, risk factors associated with fear of relapse/progression, and its impact on the lives of individuals with MS.

## 2 Materials and methods

We followed the Preferred Reporting Items for Systematic Reviews and Meta-Analyses extension for Scoping Reviews (PRISMA-ScR) guidelines (19).

### 2.1 Search strategy

We conducted a systematic search across three major databases—PubMed, Web of Science, and Scopus from inception through October 6, 2024. The search strategy was designed to capture studies focusing on fear of progression or relapse among patients with MS. The keywords used to conduct this search were: (Multiple Sclerosis) AND (“Fear of Progression” OR “Fear of Relapse” OR “Fear of Recurrence”). The search syntax for each database is provided in **Table 1**.

**Table 1:** Search strategy by database.

Database	Search syntax
PubMed	(“Multiple Sclerosis”[Title/Abstract]) AND (“Fear of Progression”[Title/Abstract] OR “Fear of Relapse”[Title/Abstract] OR “Fear of Recurrence”[Title/Abstract])
Scopus	TITLE-ABS-KEY(“Multiple Sclerosis”) AND TITLE-ABS-KEY(“Fear of Progression” OR “Fear of Relapse” OR “Fear of Recurrence”)
Web of Science	TS=(“Multiple Sclerosis”) AND TS=(“Fear of Progression” OR “Fear of Relapse” OR “Fear of Recurrence”)

All retrieved articles were imported into EndNote version 9 reference management software. Duplicates were identified and removed using the software’s automated deduplication feature, followed by manual verification to ensure accuracy.

### 2.2 Eligibility criteria

Any original paper investigating fear of progression or relapse among MS patients was considered eligible. Non-original, non-English and non-related papers were excluded from the review. The eligible studies were chosen following the PICO framework, which defines the population, independent variable, and outcome criteria (20).

o Population: People living with MS. No limitations regarding age, gender, disease severity, and the time of onset or diagnosis were considered.
o Intervention/Exposure: Fear of disease progression or relapse.
o Comparator: No limitations were considered regarding the presence of a control group.
o Outcome: Prevalence of fear and Quality of life, and any mental or physical health-related outcome measures.

Additionally, we examined risk factors for fear of relapse/progression among people living with multiple sclerosis and interventions that can mediate this fear.

### 2.3 Study selection

The lead (AM) and second author (TF), independently conducted an initial screening of the papers by reviewing the titles and abstracts, removed the duplicates, and identified relevant studies. Next, they (AM and TF) independently reviewed the full texts of the articles and assessed for eligibility based on the predefined inclusion and exclusion criteria. Collaborative discussion took place in case of a disagreement in inclusion of the studies until consensus was reached. If disagreements persisted, a third author (MFK) was consulted to provide a final decision.

### 2.4 Data extraction

The following data were extracted from each included article using Microsoft Excel (2022, version 16.62 for macOS/Windows): Senior author, publication date, country, design, scales of exposure, study subjects and their main characteristics (age, gender, type, severity of MS), main findings, and limitations. A data extraction form, designed by the lead author (AM), was utilized to ensure consistency and standardization. The form was explained to all authors for clarity prior to data collection. Two authors (AM, TF) independently extracted data from each study. To ensure accuracy, the independently extracted data were compared and discrepancies were collaboratively resolved through discussion.

### 2.5 Assessment of study quality

The risk of bias in the included studies was evaluated using the Standard Quality Assessment Criteria for Evaluating Primary Research Papers from a Variety of Fields by Kmet et al. (21). This tool offers distinct guidelines for assessing the quality of quantitative and qualitative studies. For this systematic review, only the quantitative assessment framework was applied, which consists of 14 specific criteria designed to evaluate the internal validity of studies by minimizing potential biases in their design, execution, and analysis.

Each criterion was rated based on predefined guidelines: “Yes” (score of 2), “Partial” (score of 1), “No” (score of 0), or “Not Applicable” (excluded from the calculation). These scores were then measured to produce a composite quality score for each study, ranging from 0 to 1.0, where scores above 0.75 are typically considered high quality (21, 22). All quality assessments were performed through a double-coding process to ensure reliability, with discrepancies resolved by consensus. Full guideline on the risk of bias method can be found https://ualberta.scholaris.ca/items/b8ff8755-6efb-4fb2-941a-def0d418fd07.

### 2.6 Analysis

Due to the heterogeneity of the methods, scales, and outcomes investigated in the included studies, a meta-analysis was not feasible. Thematic analysis was performed using Braun and Clarkes 6 phase thematic analysis method (23). This method involves systematically identifying, organizing, and interpreting patterns or themes within qualitative data. Two authors (AM, TF) generated the themes after familiarization with the data of the 13 studies included in this review.

The examined themes include: Scales utilized to assess fear of relapse/progression, Prevalence of fear of relapse/progression, Risk factors of fearing relapse/progression, Consequences of fear on patients’ lives, and Mediators of fear of relapse/progression.

## 3 Results

**Figure 1:**
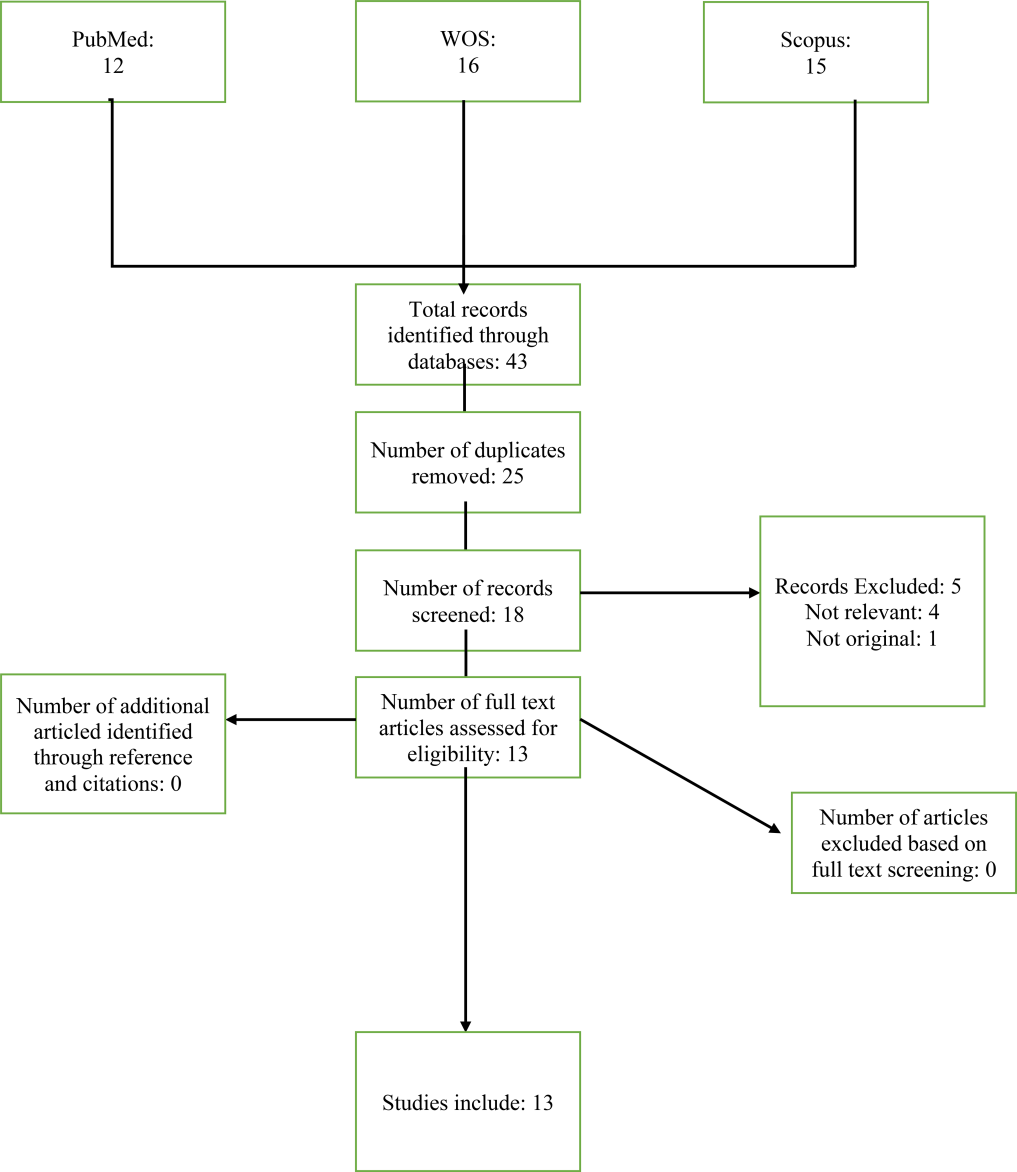
Study flow chart.

Our search strategy yielded 43 studies (**Figure 1**). After removing duplicates (n=25) and screening the titles, abstracts, and full-text articles, 13 studies met the eligibility criteria of this systematic scoping review (10, 14, 24–34). Full details of excluded studies, including titles, authors, and reasons for exclusion, are provided in the **Supplementary Table 1**.

All included studies scored above the conservative threshold of 0.75, indicating high methodological quality with minimal risk of bias (**Table 2**).

**Table 2:** Characteristics of the included studies.

Author (s) name, year of publication, country	Aim of the study	Method	Study population	Participants age (mean ± SD)	Gender distribution	Disease type	Disease duration in years (mean ± SD)	Scales utilized	Main findings	Limitations	Risk of Bias Score
Azami-Aghdash et al. 2019, Iran (24)	To examine how fear of disease progression impacts quality of life and symptoms of depression and anxiety	Cross-sectional study	70 MS patients	51.50 ± 14.80	F: 32 (45.7%) M: 38 (54.3%)	Not mentioned	5.65 ± 2.95	Fear of Progression Questionnaire-Short Form, World Health Organizations Quality of Life-Brief, Beck Depression Inventory, and Beck Anxiety Inventory.	Mean fear of progression score: 28.68 ± 9.18 A significant negative relationship between fear of disease progression and quality of life (rs= −0.53), symptoms of depression (rs= −0.52), and anxiety (rs= −0.48). (all p<0.005). Female gender was associated with [effect size] higher levels of fear of disease progression (P=0.021).	Correlational design, did not consider disease severity, population not adequately described	0.82
Broche-Pérez et al. 2022, Argentina, Mexico, Dominican Republic, Chile, Spain, Cuba, Colombia, Uruguay, Paraguay, Peru, and El Salvador (25)	To investigate the validity and reliability of the 10-item Connor-Davidson Resilience Scale (CD-RISC) among Spanish speaking MS patients	Cross-Sectional study (Online survey)	214 MS patients	40.92 ± 11.17	F: 168(78.5%) M:46(21.5%)	179 RRMS 17 PPMS 14 SPMS 4 PRMS	7.06 ± 7.67	CD-RISC 10, Multiple Sclerosis Quality of Life 29 items version, Fear of Relapse Scale, Modified Fatigue Impact Scale 5-item version	Fear of relapse total score: 40.82 ± 17.45 (mean, SD) There was a significant inverse correlation between fear of relapse scores and resilience scores (r=-0.327), quality of life (r=-0.451), Physical health composite of QOL (r=-0.212), Mental Health composite of QOL (r=-0.520), and a positive correlation with fatigue (r=0.577).	Heterogenicity of the phenotypes investigated, Higher percentage of women (78.5%) compared to men	0.91
Broche-Pérez et al. 2022, Argentina, Mexico, Uruguay, Dominican Republic, Spain, Cuba, Colombia, Guatemala, Chile, Paraguay, Peru, and El Salvador (26)	To investigate the psychometric properties of the Spanish version of the Fear of Relapse Scale in MS patients and examine the association between the Fear of Relapse Scale and fatigue, as well as perceived cognitive deficits	Cross-Sectional study (Online Survey)	173 MS patients	39.60 ± 10.78	F: 145 (83.8%) M: 28 (16.2%)	All RRMS	6.26± 6.84	Fear of relapse scale, Modified Fatigue Impact Scale 5-item version, Perceived deficits questionnaire 5-item version	Fear of relapse (Total score): 40.71 ± 18.04 Factor 1 (Fear of disability): 21.04 ± 10.63 Factor 2 (Fear of the psychological and physiological consequences):14.21 ± 5.82 Factor 3 (Limitations resulting from fear): 5.44 ± 3.91 Fear of relapse correlated with self-perceived health (r=-0.342), fatigue (r=0.455) and cognitive deficits (r=0.314). Fatigue explained 31.8% of the variance in fear of relapse. _	The divergent validity of the Fear of Relapse Scale was not examined. Did not investigate the associations between fear of relapse and disease duration, degree of disability, or number of relapses.	0.82
Broche-Pérez et al. 2023, Argentina, Guatemala, Mexico, Dominican Republic, Chile, Spain, Cuba, Colombia, Uruguay, Paraguay, Peru, and El Salvador (27)	To examine how psychological resilience can mediate the relationship between fear of relapse and quality of life	Cross-Sectional study (Online Survey)	240 MS patients	40.62 ± 11.15	F: 192 (80%) M: 48 (20%)	179 RRMS 17 PPMS 14 SPMS 4 PRMS 26 unknown	6.86 ± 7.51	Multiple Sclerosis Quality of Life 29 items version, Fear of Relapse scale, Connor-Davidson Resilience Scale-10	Fear of relapse (total score): 40.93 ± 17.82 Significant inverse correlations between fear of relapse and quality of life (-0.44) and resilience (-0.23). Psychological resilience had a small protective mediating effect on the relationship between fear of relapse and quality of life (β = -0.36 was reduced by -0.041, 95% CI [-0.084, -0.010] with the addition of the mediator).	Cross sectional design, heterogeneity of the phenotypes included, did not consider other variables that may influence the quality of life in people with MS including fatigue levels, depression, anxiety, cognitive functioning, and tolerance to uncertainty.	0.82
Ebrahimi et al. 2023, Iran (28)	To explore the impact of collaborative care on the fear of disease progression in MS patients	Randomized control trial	79 patients (36 patients and their caregivers in the intervention and 43 in the control group)	38.22 ± 9.24	F: 69 (87.3%) M: 10 (12.7%)	54 CIS 12 RRMS 3 PPMS 10 SPMS	NM	Fear of Progression Questionnaire-short form	Fear of progression score decreased from 37.80 ± 12.22 to 34.86 ± 12.21 (P=0.001) in the intervention group and increased from 38.39 ± 11.41 to 40.20 ± 11.68 (P=0.001) in the control group. Small marginally significant group by time interaction (P=0.051).	Intervention was insufficiently described, conducting the study during the COVID-19 pandemic could affect external validity.	0.82
Hosseinchi et al. 2022 Iran (29)	To explore biased interpretation of ambiguous bodily information among patients with MS	Cross-sectional	65 MS patients, 65 controls	37.5 ± 7.28	F: 36 (55.38%) M: 29 (44.61%)	All RRMS	NM	Fear of relapse scale, Health Anxiety Inventory -18	Fear of relapse scores: 60.92 ± 25.36 MS patients showed higher negative interpretation bias (12.32 ± 2.44 *vs*. 4.55 ± 2.30) and health anxiety (32.08 ± 12.84 *vs*. 13.05 ± 7.80) than controls (P < 0.001). Negative interpretation bias correlated strongly with health anxiety (r = 0.75, P < 0.001) and fear of relapse (r = 0.67, P < 0.001). Fear of relapse also correlated with health anxiety (r = 0.67, P < 0.001). Health anxiety partially mediated the relationship between negative interpretation bias and fear of relapse (indirect effect: r = 0.297, P < 0.001)	Separate recruitment of case and control group, did not consider general anxiety as a comorbid condition, the scenarios included in the study were general health-related and not specific to complications related to RRMS	0.77
Khatibi et al. 2020, Iran (10)	To establish the psychometric properties of a new assessment of fear of relapse in RRMS patients.	Interview/Cross-sectional/Longitudinal	33 MS patients for the development of the instrument 168 MS patients for psychometric evaluation	Interview group: 33.18 ± 6.2 Psychometric group: 32.65 ± 8.2	Interview group: F: 18 (54.54%) M: 15 (45.45%) Psychometric group: F: 142 (84.52%) M: 26 (15.47%)	All RRMS	Interview group: 6.71 ± 3.3 Psychometric group: 3.19 ± 1.5	Fear of Relapse Scale, Depression Anxiety Stress Scale (DASS-21), Intolerance of Uncertainty-27	The scale showed evidence of validity and reliability Fear of relapse (total score): 41.6 ± 21.1 No relationships were found between fear of relapse and disease duration, number of relapses, or time since relapse.	Varying disease duration of patients, varying disease phenotypes	0.82
Khatibi et al. 2021, Iran (14)	To explore the role of fear of relapse in health-related quality of life in MS patients	Cross-sectional	70 MS patients 70 controls	33.69 ± 7.8	F: 44 (62.85%) M: 26 (37.14%)	All RRMS	8.24 ± 5.6	HRQoL-36 (short form), Health Anxiety Inventory -18, Fear of Relapse scale	Mental and physical quality of life was significantly lower for MS patients than controls (53.09 ± 21.1 *vs* 70.50 ± 12.3 and 53.09 ± 21.1 *vs* 70.31 ± 11.7, respectively). Multivariable linear regression model (R² = 0.61, P < 0.001) identified fear of relapse as the strongest predictor of physical HRQoL in RRMS patients (β = -0.70, P < 0.001). Adding fear of relapse to the model substantially decreased the strength of the relationship between disease severity and quality of life (β = -0.63, P<.001 reduced to β = -0.30, P=0.08). Health Anxiety was significantly associated with fear of relapse (β = 0.27, R² = 0.07, P=0.026)	Cross-sectional design, recall bias, limited access to medical records, selection bias, groups recruited from different settings	0.91
Moghadasi et al. 2023, Iran (30)	To investigate fear of relapse, reinfection, and anxiety during COVID-19 pandemic in MS patients	Cross-sectional	368 MS patients	37.4 ± 8.7	F: 299 (81.3%) M: 69 (18.8%)	All RRMS	7.7 ± 5.5	Expanded Disability Status Scale, Fear of Relapse Scale, Beck Anxiety Inventory	Fear of relapse: 49.7± 20.5 43% were afraid of experiencing a relapse triggered by COVID-19 infection. 14.4% of patients reported a relapse during their most recent COVID-19 infection. Higher anxiety score was associated with fear of relapse (rho = 0.49). Patients with a history of relapse during COVID-19 had significantly higher fear of relapse scores than those that did not (58.8 ± 24.0 *vs* 48.1 ± 19.4)	Did not obtain disease modifying therapies of patients, conducting the study during the COVID-19 pandemic could affect external validity. Self-report of MS relapse.	0.82
Nickel et al. 2017, Germany (31)	To explore how MS patients assess their HRQoL and identify the sociodemographic and disease-related factors that influence HRQoL	Cross-sectional	1220 MS patients	47.8 (SD not mentioned)	F: 927 (76%) M: 293 (24%)	59% RRMS 12% PPMS 21% SPMS 8% Unknown	11.5 (SD not mentioned)	Multiple Sclerosis International Quality of Life (MusiQoL), 12-items short form of the Fear of Progression Questionnaire	Fear of progression (mean, SD): 2.66± 0.78 Most frequent fears were dependency (39.6%), losing hobbies (38.4%), and medication side effects (33.8%). Disease severity and comorbidity explained 14% to 48% of the variance across domains of the MusiQoL and 17% of the variance in fear of progression.	Immobile patients were overrepresented, cross-sectional design, elimination of items in four original domains of the MusiQoL scale, self-report of disease severity and comorbidities	0.91
Nielsen et al. 2022, Germany (32)	To determine the key aspects of wisdom and self-management strategies that help individuals with MS cope with the fear of disease progression	Cross-sectional	92 MS patients	47.87 ± 8.94	F (61)66.3% M (31) 33.7%	72.8% RRMS 4.4% PPMS 13% SPMS 1.1 % CIS 8.7% unknown	10.84 ± 8.52	Self-Assessed Wisdom Scale, Questionnaire to Assess Resources and Self-management Skills, Fear of Progression Questionnaire, Fatigue Scale for Motor and Cognitive Functions, Center for Epidemiologic Studies Depression Scale, Cognitive Reserve Index	Fear of progression sum score: 10.99± 3.10 Younger patients had higher fear of progression scores (*r*=)? Managing emotions (as part of wisdom), self-observation and self-efficacy (as self-management skills), and handling fatigue are key elements in addressing certain aspects of fear of progression	Cross-sectional design, use of self-reports	0.86
Özden et al. 2022, Turkey, (33)	To translate the Fear of Relapse Scale into Turkish, adapt it for cultural relevance, and evaluate its psychometric properties.	cross-sectional and prospective study	101 MS patients	37.6 ± 10.0	F 82 (81.2%) M 19 (18.8%)	Not mentioned	6.7 ± 5.3	Fear of Relapse scale (translated to Turkish and culture cross adopted), Depression Anxiety Stress Scale (DASS-21), Intolerance of Uncertainty (IUS-12)	Fear of relapse (total score): 33.7 ± 16.5 Fear of relapse correlated significantly with DASS-21 depression (r=0.641), anxiety (r=0.648), stress (r=0.631), and IUS-12 (r=0.609). The Turkish version of the fear of relapse scale has _[number]: test-retest reliability	Did not analyze the questionnaires responsiveness, heterogeneous sample of MS patients, did not explore convergent validity with fear of falling or other disease-specific neuropsychiatric changes	0.91
Shaygannejad et al. 2021 Iran (34)	To investigate fear of relapse, psychological well-being, and social support during the COVID-19 pandemic	Cross-sectional	165 MS patients	35.3 ± 8.6	F 136 (82.4%) M 29 (17.6%)	All RRMS	7.1 ± 5	DASS-21 Persian version, Fear of Relapse scale, perceived social support questionnaire	Average scores: social support 63.1 ± 16.8, DASS-21 16.4 ± 13.4, and Fear of relapse 51.4 ± 17.3. Social support reduced fear of relapse (r=-0.25), while depression (0.47), anxiety (0.53), and stress (0.54) increased fear of relapse. Sex was an independant predictor of fear of relapse	Cross-sectional design. Conducting the study during the COVID-19 pandemic could affect external validity	0.82

HRQoL, health-related quality of life; CIS, Clinically isolated syndrome; RRMS, Relapse remitting MS; PPMS, Primary Progressive MS; SPMS, Secondary Progressive MS; M, Males; F, Females; NM, Not Mentioned.

### 3.1 Study features

Characteristics of the included studies are demonstrated in **Table 2**. Papers published between 2017 to 2023 and were conducted Argentina, Mexico, Dominican Republic, Chile, Spain, Cuba, Colombia, Uruguay, Paraguay, Peru, El Salvador, Iran, Turkey, and Germany. Of the 3058 individuals enrolled across all studies, 76.88% were women and 23.11% were men. Mean age of the participants in the studies ranged from 33.18 to 51.5 years old.

### 3.2 Scales utilized to assess fear of MS progression/relapse

The Fear of Relapse Scale developed by Khatibi et al. in 2020 (10) was employed by 9 out of the 13 studies included in this review (10, 14, 25–27, 29, 30, 33, 34). This scale has been validated in multiple languages, including English, Persian, Turkish, and Spanish (10, 26, 33). The Fear of Relapse Scale comprises 26 items categorized into three dimensions: Fear of disability following a relapse (13 items), Fear of the psychological and physical effects of a relapse (8 items), and Limitations due to fear (5 items). Each item is rated on a five-point Likert scale, ranging from 0 (never) to 4 (always). The scale has demonstrated strong psychometric properties, including a high internal consistency (Cronbach’s alpha = 0.92) and reliability (test-retest correlation = 0.74).

One study utilized the Fear of Progression Questionnaire, a 43-item self-report tool designed to assess fears related to disease progression across five subscales: affective reaction, partnership/family, occupation, loss of autonomy, and anxiety coping. Responses are rated on a five-point Likert scale (32). This scale is validated and widely used to study the impact of many different diseases, including MS, diabetes, and cancer and has shown high internal consistency (Cronbach’s alpha = 0.95) and high test-retest reliability over one week (rtt = 0.94) (11, 32, 35). The remaining three studies (24, 28, 31) utilized a shorter 12-item version of the Fear of Progression Questionnaire, which offers a more concise assessment of these same five dimensions. The scale’s reliability (Cronbach’s α > 0.7) and validity (coefficients ranging from 0.77 to 0.94) has been established. (28, 36).

### 3.3 Fear of progression

The four studies that examined fear of disease progression using the Fear of Progression Questionnaire (or its short form) revealed significant variability in levels of concern between individuals. One study that used the full form of the questionnaire reported that 18.4% of patients had low fear of progression, 61.9% moderate fear, and 19.6% dysfunctional levels of fear (32). The remaining three studies that utilized the short form of the Fear of Progression Questionnaire demonstrated that a significant proportion of patients expressed concerns about the progression of their disease (24, 28, 31). The most commonly reported fear was reliance on external help for daily activities, with nearly 40% of participants indicating they often or very often experienced this fear, followed by fear of inability to pursue hobbies and potential harm from medication (31).

### 3.4 Fear of relapse

The Fear of Relapse Scale, specifically designed for Relapsing-Remitting MS (RRMS) populations, consistently highlighted significant concerns among patients. The total mean fear of relapse scores ranged between 33.7 ± 16.5 (33) in Turkish-speaking patients to 60.92 ± 25.36 in studies conducted in Iran (29), with variability observed across geographic and linguistic groups. Turkish-speaking patients demonstrated the lowest scores (33.7 ± 16.5), reflecting generally mild to moderate levels of concern. Scores from Spanish-speaking countries ranged narrowly from 40.71± 18.04 to 40.93 ± 17.82 (25–27). Studies from Iran revealed a broader range of higher scores of 41.6± 21.1 (10), 49.7± 20.5 (30), 51.4± 17.3 (34), and 60.92± 25.36 (29), reflective of mild to very high levels of concern. Spanish-speaking MS patients reported a higher level of concern about psychological and physiological consequences (mean score per item: 1.78) compared to their concern about the risk of disability (mean score per item: 1.62) or limitations due to fear (mean score per item: 1.09) (26). Iranian MS patients (10) most feared worsening fatigue and the psychological impact of receiving grave news.

### 3.5 Risk factors of fear of relapse/progression

Two demographic factors were identified as significant contributors to fear of relapse or disease progression in patients with MS. Women were found to experience higher levels of fear of disease progression compared to men (24), with sex being identified as an independent predictor of this fear (34). Younger patients also reported higher fear of progression (32).

The relationship between disease characteristics and fear of relapse was less consistent. While no correlation was found between fear of relapse and disease duration, number of relapses, or time since the last relapse (10), weak associations suggested that fear of progression might increase slightly with comorbid conditions and decrease with longer disease duration (31).

Psychological factors played a significant role in predicting fear of relapse or progression. Fear of relapse was strongly associated with biased interpretations of ambiguous bodily information (29) (r = 0.75) and with perceived cognitive deficits (r=0.314), suggesting that negative cognitive biases might amplify fears about future relapses. Intolerance of uncertainty, which measures people’s reaction to uncertainties in life (37), was another factor that independently correlated with fear of relapse (10, 33) (r= 0.52, r=0.61, respectively). Fatigue also emerged as a critical factor influencing fear of relapse (r=0.455, β=0.318) (26). Higher fatigue levels were also associated with the “loss of autonomy” subscale in the fear of progression questionnaire (β=0.26) (32). The COVID-19 pandemic provided additional insight into how social/environmental factors might influence fear of relapse; patients who experienced a relapse during the pandemic reported significantly higher fear of future relapse compared to those who did not (58.8 ± 24.0 *vs*. 48.1 ± 19.4) (30). Greater social support was inversely correlated with this fear (r=-0.25) (34).

### 3.6 Fear of disease relapse/progression impacts health/wellness

Numerous studies have demonstrated that fear of disease progression and relapse is correlated with depression, anxiety, stress, and diminished psychological well-being (14, 24, 25, 29, 30, 33, 34) (See **Table 2** for effect sizes). Increased fear adversely affected quality of life among people living with MS (β = -0.70, r= −0.53, r=-0.451, respectively) (14, 24, 25). Notably, in RRMS patients, fear of relapse emerged as the strongest predictor of quality of life, surpassing other factors such as age, education level, disease characteristics, and neurologists’ estimates of disease severity (14). Those with greater fear of relapse also perceived their health more poorly overall (r=-0.342). The three factors of the fear of relapse scale—fear of disability, psychological consequences, and limitations from fear—were all independently associated with poorer health self-perception (26).

### 3.7 Psychological factors influence fear of relapse and quality of life

Nielson and colleagues found that the following aspects of psychological resilience were determinants of fear of progression: emotional regulation (β= -0.26), self-observation (β=0.27, 0.26), and self-efficacy (β=-0.35) (32).Broche-Perez et al. showed that psychological resilience could also slightly reduce the adverse impact of fear of relapse on quality of life (including psychological resilience as a mediator reduced the β coefficient from -0.36 to -0.32) (27).

### 3.8 Interventions targeting fear of relapse/progression

Only one randomized controlled trial examined the impact of a 3-month nurse/physician collaborative care intervention on fear of progression in MS. The intervention encouraged patients to actively engage in managing their condition through seven 90–120 minute educational sessions, collaborative follow-up meetings (“in two collaborative care training sessions in the third and fourth weeks and two follow-up visits, with two weeks apart and the second training session in 90–120 minutes”), and periodic assessments. (38).

These sessions of collaborative care over nine weeks slightly decreased fear of progression scores from 37.80 ± 12.22 to 34.86 ± 12.21, whereas those who did not undergo collaborative care showed a small increase in fear (38.39 ± 11.4 to 40.20 ± 11.68). Between-group differences were small and not statistically significant (28).

## 4 Discussion

This systematic review synthesized current literature on the prevalence, impact, and associated factors of fear of relapse and disease progression among individuals living with MS. In each subsection below, we delve into the implications of these findings, compare them with existing literature, acknowledge the limitations of our review, and propose recommendations for clinical practice and future research.

### 4.1 Prevalence and impact of fear in MS

The studies consistently show that fear of relapse and disease progression is a frequent and substantial concern among people with MS. This aligns with findings in other chronic diseases—such as cancer, diabetes, and rheumatoid arthritis—where fear of disease progression is recognized as a common and distressing issue (39–41).

Our review found robust associations between fear of relapse/progression and several negative psychological outcomes, such as depression, anxiety, stress, fatigue, health anxiety (10, 14, 24–26, 29, 30, 32–34), and poor quality of life (14, 25, 26, 34). These findings suggest that fear of relapse/progression is not an isolated concern but part of a broader psychological profile, creating a vicious cycle in which psychological distress heightens fear, further worsening mental health and quality of life.

The high prevalence and significant impact of fear of relapse/progression necessitate proactive clinical interventions. Clinicians should routinely screen for fear using validated tools like the Fear of Relapse Scale or the Fear of Progression Questionnaire. Early identification allows for timely psychological support and tailored interventions. Screening is particularly important for women and younger individuals who experience greater fear of relapse/progression on average (24, 34). They particularly fear becoming dependent on others for daily activities (31) during a time in their lives where there is likely a greater societal expectation to be productive and to care for others. Previous studies investigating fear of recurrence in cancer have also demonstrated that younger individuals were more likely to be affected by this fear (17).

### 4.2 Clinical predictors and potential interventions

Clinical variables such as disease duration/severity, number of relapses, and time since the last relapse were not consistently associated with fear of relapse/progression (14). In contrast, social support, psychological resilience, and individual perceptions of one’s own health were much more influential (26, 27, 34). Interpreting ambiguous bodily stimuli as potential symptoms, experiencing more fatigue, and perceiving cognitive impairment significantly contributed to fear of relapse (29).

Exercise is a prime community-based intervention that addresses many factors linked to increased fear of progression; it concurrently reduces MS fatigue (42), enhances interoception (43), and may provide opportunities for social contact. Future in-clinic interventions educating patients about benign bodily sensations (29), guiding them towards reliable information sources, and cautioning against misinformation could also empower patients to interpret interoceptive signals more accurately and reduce unnecessary anxiety and fear. In support of this, a collaborative nursing/physician educational/monitoring intervention did produce a small positive impact (28). It is likely that integrating psychological services into MS care could provide further benefit through targeted stress management and resilience training. However, it is important to highlight that current evidence on interventions specifically targeting fear of relapse or progression remains extremely limited. To date, only one interventional study (28) has directly addressed this outcome.

Consistent with the broader literature emphasizing the importance of social networks for managing chronic illness (44), social support is associated with lower levels of fear of progression. Group- and community-based interventions may therefore be especially beneficial (34). Encouraging collaboration and interaction between newly diagnosed MS patients and those with well-controlled long-term disease could help alleviate the fear of relapse by providing reassurance and practical coping strategies. Such peer support initiatives have shown promise in previous studies (45, 46). Social workers, support groups, and/or recreational therapists can link patients to community programs and supportive resources.

The aim of any intervention targeting fear of relapse or progression should not be to eliminate the fear entirely, but to harness it as a motivator for self-care (47) while providing coping skills and resources to manage an uncertain disease course. Collaborative care models involving multidisciplinary teams—including neurologists, psychologists, social workers, exercise scientists, and patients with similar conditions—are likely needed to provide comprehensive support.

### 4.3 Limitations

The included studies were mostly conducted in Iran, Turkey, Germany, and Spanish-speaking countries, which limits the generalizability of findings to other regions with different cultural perceptions of illness and different health care systems. Two different assessment tools were used (Fear of Relapse Scale and Fear of Progression Questionnaire) with some differences in their focus and scope. Substantial heterogeneity in MS types (e.g., RRMS, PPMS, SPMS), disease durations, and severity levels further complicates synthesis of the evidence. Most studies employed cross-sectional designs, precluding causal inferences. While these studies provide valuable insights into associations between fear of relapse or disease progression and psychological or clinical outcomes, they do not allow for conclusions about causality or temporal sequence. As a result, it remains unclear whether fear contributes to increased anxiety, fatigue, and reduced quality of life, or whether these factors, in turn, exacerbate fear.

Another limitation was that our search was limited to PubMed, Scopus, and Web of Science, which may have led to the omission of relevant studies indexed in other databases such as EMBASE or Cochrane.

Finally, the potential for publication bias exists, as studies reporting significant findings are more likely to be published.

### 4.4 Future research directions

Future studies should aim to address these limitations. Conducting research in diverse geographic and cultural contexts will enhance the generalizability of findings. Cultural context likely plays a significant role in shaping how individuals perceive and respond to fear of relapse or disease progression, yet this aspect remains underexplored in the current literature. Most included studies were conducted in Iran, Turkey, Germany, and Spanish-speaking countries, each with distinct cultural beliefs, health system structures, social support norms, and attitudes toward chronic illness. For example, societies with strong family-oriented support systems may buffer some of the anxiety associated with disease progression, while cultures with high stigma around disability or chronic disease could amplify fear and psychological distress (48). Differences in health literacy, trust in healthcare providers, and access to reliable information may also influence how bodily changes are interpreted and whether they are perceived as threatening. Consequently, findings from these regions may not fully capture the experiences of individuals living with MS in other cultural contexts, such as North America. Future research should explicitly investigate how cultural values, beliefs, and healthcare contexts shape the experience of fear and develop culturally sensitive interventions tailored to diverse populations.

Longitudinal observational designs can track changes in fear throughout the disease course and establish directionality of associations between fear and clinical outcomes. Intervention studies are particularly needed. Randomized controlled trials evaluating the efficacy of psychological interventions—such as cognitive-behavioral therapy, resilience training, and social support enhancement—should add fear of progression/relapse as an outcome variable or mediator variable. To ensure health equity in regions with limited access to specialized care, research on digital health interventions, such as telemedicine counselling or online support groups, is especially high priority.

Qualitative studies exploring patients’ lived experiences would deepen understanding of the nuances of fear in MS and inform intervention development/implementation.

## 5 Conclusion

Fear of relapse and disease progression has been frequently reported among individuals with MS and may negatively influence psychological well-being, contributing to higher levels of anxiety, depression, stress, fatigue, and reduced quality of life. Available studies suggest that psychosocial and perceptual elements, including levels of social support, resilience, perceptions of health, and interpretation of bodily signals, may play a more prominent role in shaping this fear than clinical characteristics alone. However, the limited number of studies—particularly the scarcity of interventional research—makes it difficult to draw firm conclusions about its prevalence, predictors, or clinical consequences. These uncertainties highlight the need for routine assessment of fear in clinical practice for example using the Fear of Relapse Scale (specific to RRMS) or the Fear of progression questionnaire (applicable across chronic illnesses), and for future research to design and rigorously evaluate targeted interventions such as psychoeducation, resilience training and peer support.
